# 胸腺素β10在人肺腺癌细胞株A549中抑制细胞凋亡、促进细胞增殖机制研究

**DOI:** 10.3779/j.issn.1009-3419.2014.11.03

**Published:** 2014-11-20

**Authors:** 紫璇 李, 连悦 曲, 红珊 钟, 克 徐, 雪杉 邱

**Affiliations:** 1 110001 沈阳，中国医科大学附属第一医院放射科，辽宁省影像诊断与介入治疗重点实验室 Department of Radiology and Key Laboratory of Diagnostic Imagingand Interventional Radiology, the First Affiliated Hospital of China Medical University, Shenyang 110001, China; 2 110001 沈阳，中国医科大学附属第一医院病理科，基础医学院病理学教研室 Department of Pathology, the First Affiliated Hospital of China Medical University and College of Basic Medical Sciences, China Medical University, Shenyang 110001, China; 3 110001 沈阳，中国医科大学附属第一医院药学部 Department of Pharmacy, the First Affiliated Hospital of China Medical University, Shenyang 110001, China

**Keywords:** 肺肿瘤, Tβ10, 增殖, 凋亡, Lung neoplasms, Thymosin β10, Proliferation, Apoptosis

## Abstract

**背景与目的:**

胸腺素β10（thymisin β10, Tβ10）是胸腺素家族的成员之一，它的分子量在5 kDa左右，是哺乳动物体内含量最丰富的β胸腺素之一，作为一种肌动蛋白结合蛋白，它可能通过调控肌动蛋白的结构改变细胞的生长、死亡、粘附和迁移。Tβ10在肿瘤的增殖、凋亡、血管形成方面也发挥重要的作用。然而Tβ10在不同类型的肿瘤中所发挥的作用有很大差异且Tβ10对肺癌细胞增殖和凋亡的影响尚未见文献报道。本研究选择肺腺癌细胞系A549作为研究对象，通过加入Tβ10或用小干扰RNA干扰Tβ10的方法，检测肺癌细胞凋亡、增殖及细胞周期的变化，探讨Tβ10对肺癌细胞这几种生物学行为的影响及其可能的机制。

**方法:**

流式双染检测加入Tβ10或干扰Tβ10后细胞凋亡的变化，PI染色后检测细胞周期的变化，CCK-8法检测细胞增殖能力的变化，Real-time PCR及蛋白免疫印迹检测增殖、凋亡相关基因的变化。

**结果:**

加入Tβ10能抑制A549细胞的凋亡，促进细胞的增殖，增加S期和G_2_期/M期细胞的比率，减少Caspase-3、P53表达的同时增加Cyclin A、Cyclin E表达；干扰Tβ10能促进A549细胞的凋亡，抑制细胞的增殖，增加G_0_期/G_1_期细胞的比率，增加Caspase-3、P53表达的同时减少Cyclin A、Cyclin E表达。

**结论:**

在肺癌细胞系中Tβ10能够通过抑制P53的表达抑制细胞凋亡，能够通过上调Cyclin A、Cyclin E的表达水平，促进细胞周期进程，促进细胞的增殖。Tβ10可能成为肺癌诊断的分子标记物及治疗靶标。

## Abstract (en)

**Background and objective:**

Thymosin beta 10 (Tβ10) is one of β-thymosin family members, has a highly conserved polar 5 kDa peptides. This peptide is now regarded to be a small actin-binding protein and thereby induce depolymerization of the intracellular F-actin networks. Alteration of Tβ10 expression may alter the balance of cell growth, cell death, cell attachment and cell migration. Tβ10 also affects cell metastasis as well as proliferation, apoptosis and vascularization of cancer cells. But function of Tβ10 appear to be rather different between cancer cells, and the molecular mechanisms of β-thymosins to regulate cell apoptosis and proliferation in NSCLC (non-small cell lung cancer) cell lines are unclear. In this study, we used lung adenocarcinoma cell line A549, added Tβ10 or down-regulated the expression of Tβ10. We observed the change of apoptosis, proliferation and cell cyclin ability in A549 and the mechanisms underline them were also identified.

**Methods:**

After A549 was treated with 100 ng/mL recombinant human Tβ10 or siTβ10, apoptosis rate of A549 and cell cycle distribution were detected by flow cytometry (FCM). CCK-8 assay was employed to determine the proliferation of A549. The mRNA level of P53, Caspase-3, Cyclin A and Cyclin E were determined by real-time PCR. The protein level of P53, Caspase-3, Cyclin A and Cyclin E were detected by Western blot.

**Results:**

Add Tβ10 can inhibit the apoptosis and prompt the proliferation of A549. It can also increase the cell rates of S-phrase and G_2_/M-phrase, decrease the expression of P53 and Caspase-3, but increase the expression of Cyclin A and Cyclin E. Interferance of Tβ10 can prompt the apoptosis and inhibit the proliferation of A549. It can also increase the cell rates of G_0_/G_1_-phrase, increase the expression of P53 and Caspase-3, but decrease the expression of Cyclin A and Cyclin E.

**Conclusion:**

In lung cancer cell line, Tβ10 can inhibit the apoptosis by increase P53, drive cells into the S and G_2_/M-phase, prompt cell proliferation by increase the expression of Cyclin A and Cyclin E. Tβ10 may become a potential biomarker and therapy target for non-small cell lung cancer.

近年来，肺癌的发病率和死亡率增长很快，且呈不断上升趋势，已经成为对人类健康危害最大的肿瘤^[1]^。由于很多患者确诊时已是癌症的中晚期，肺癌的5年生存率依然很低^[2]^，因此需要对肺癌的分子生物学特点进行深入的研究。凋亡对促进机体的发育及去除老化，损伤组织方面有重要作用^[3]^。与正常细胞不同，肿瘤细胞能在有环境压力的条件下逃避凋亡，并且快速增殖。如何能促进肿瘤细胞凋亡，抑制细胞增殖是目前亟待解决的科学问题。

胸腺素β10（thymisin β10, Tβ10）胸腺素家族的成员，在人体中分布广泛。作为一种肌动蛋白结合蛋白，Tβ10在细胞的迁移过程中发挥重要作用。有文献^[3-5]^报道Tβ10在炎症反应、肿瘤的增殖、凋亡、血管形成方面发挥重要的作用。然而Tβ10在不同类型的肿瘤中所发挥的作用有很大差异。在甲状腺癌、胰腺癌、乳腺癌、结肠癌中Tβ10能够促进肿瘤血管生成和肿瘤细胞侵袭转移^[4, 6, 7]^；而在卵巢癌组织和细胞中Tβ10表达降低，并起到抑制肿瘤的生长、促进肿瘤的凋亡的作用^[8]^。我们前期的研究发现Tβ10在非小细胞肺癌（non-small cell lung cancer, NSCLC）中表达上调，并与肺癌的分期、分化、淋巴结转移和患者的预后有关^[9]^，Tβ10可能是通过上调血管内皮生长因子C（vascular endothelial growth facor-C, VEGF-C）的表达促进淋巴管的形成^[10]^。然而Tβ10对肺癌细胞凋亡及细胞周期的影响及其机制尚不清楚。

本研究选择肺腺癌细胞系A549作为研究对象，通过加入Tβ10或用小干扰RNA干扰Tβ10的方法，检测肺癌细胞凋亡、增殖及细胞周期的变化，探讨Tβ10对肺癌细胞这几种生物学行为的影响及其可能的机制。

## 1 材料与方法

### 1.1 主要试剂

人肺癌细胞系A549购自ATCC。细胞培养用DMEM高糖培养基购自Gibco，胎牛血清购自碧云天。外源Tβ10蛋白（38-43）购自Abcam，小干扰RNA购自上海吉玛，转染试剂购自Qiagen。RNA提取购自Invitrogen，反转录试剂盒、Real-time PCR试剂盒及PCR引物合成购自Takara。抗P53、Caspase-3、Cyclin A、Cyclin E单克隆抗体购自CST；β-actin抗体购自Santa Cruz；辣根过氧化物酶标记的山羊抗小鼠及山羊抗兔IgG购自中杉金桥。BCA法蛋白定量试剂盒购自碧云天，裂解液及超敏发光试剂盒购自Pierce。CCK-8凋亡检测试剂盒购自日本同仁，流式凋亡检测试剂盒购自BD。

### 1.2 细胞培养

肺癌细胞系A549使用含有10%的小牛血清DMEM培养基，37 ℃、5%CO_2_的条件下培养，每两天换一次液，并用0.25%的胰蛋白酶进行消化传代。在加入Tβ10组实验前取对数生长的细胞，饥饿4 h后加入100 ng/mL Tβ10，按时间点收集细胞。转染siNC和siTβ10组于转染后48 h收集细胞。转染序列如下：siNC：sense：5’-UUC UCC GAA CGU GUC ACG UTT-3’，anti-sense：5’-ACG UGA CAC GUU CGG AGA ATT-3’；siTβ10：sense：5’-CGA CCA AAG AGA CCA UUG ATT-3’，anti-sense：5’-UCA AUG GUC UCU UUG GUC GTT-3’。每次实验同一个处理因素设两个复孔，重复三次实验。

### 1.3 实时定量PCR

细胞提取总RNA后，反转录成cDNA，使用SYBR Green法，进行Real-time PCR扩增，总体积20 μL。扩增过程如下: 95 ℃，30 s；95 ℃，5 s；60 ℃，30 s，40个循环。β-actin作为内参。基因相对表达水平计算方式如下：ΔCt=Ct^gene^-Ct^reference^，增加倍数用2^-ΔΔCt^方法计算.每次试验均做三个重复孔。

### 1.4 细胞凋亡检测

细胞加入Tβ10 24 h或转染siTβ10 48 h后，使用PBS清洗两次，0.25%胰蛋白酶消化，用培养基终止消化后，将细胞收集到EP管中，1, 000 rpm 4 ℃离心5 min后，去上清。用PBS洗两遍后每个样本中加400 μL缓冲液，吹打成单细胞悬液，后避光加入FITC/Annexin V 10 μL和PI 5 μL染色20 min后上机检测。

### 1.5 细胞增殖能力实验

将转染24 h后的单个细胞悬浮在96孔培养板中，密度为5×10^3^个/100 μL/孔，加入Tβ10组于检测前24 h加入Tβ10，检测前4 h每孔加入10 μL Cell Counting Kit-8®继续培养。检测时在450 nm波长下测定各孔光吸收值，以不含细胞的等体积培养基作对照。绘制细胞生长曲线。

### 1.6 细胞周期检测

收集待检测细胞用冰PBS清洗两次，75%乙醇固定30 min，固定好的细胞使用冰PBS清洗两次，使用300 μL PI避光染色30 min，流式细胞仪每个样本抽取10, 000个细胞，处于G_0_期/G_1_期、S期和G_2_期/M期的细胞分别被计数。

### 1.7 Western blot法检测蛋白表达

收集细胞并加入裂解液充分裂解，低温高速离心（4 ℃，12, 000 rpm/min，30 min），提取上清为总蛋白。每个泳道加入总蛋白60 μg，12%SDS-PAGE凝胶电泳，转印（60 V，120 min）到PVDF上。5%牛血清白蛋白（BSA）室温封闭2 h。抗P53、Caspase-3、Cyclin A、Cyclin E和β-actin（1:1, 000）4 ℃孵育过夜。分别与对应的二抗（1:2, 000）室温孵育2 h，ECL显色，结果经自动电泳凝胶成像分析仪采集，进行灰度测定。

### 1.8 统计学方法

采用SPSS 19.0统计软件，采用*t*检验进行数据分析，数据用均值±标准差（Mean±SD）表示，以*P*＜0.05为差异有统计学意义。

## 2 结果

### 2.1 Tβ10对A549细胞凋亡的影响

在A549细胞中加入100 ng/mL Tβ10，在对照组加入同体积的PBS，24 h后使用流式细胞仪检测，结果显示：加PBS组细胞凋亡率为（5.70±0.27）%，而加入100 ng/mL Tβ10组细胞凋亡率为（2.87±0.12）%，差异具有统计学意义（*P*＜0.01）。随后我们使用了Tβ10的小干扰RNA，在保证了转染效率的前提下（图 1B），检测两组的凋亡水平变化。转染siNC组细胞凋亡率为（4.25±0.24）%，而转染siTβ10组细胞凋亡率为（13.11±0.49）%，差异也具有统计学意义（*P*＜0.01）（图 1）。以上结果说明Tβ10可以抑制A549细胞的凋亡。

**1:**
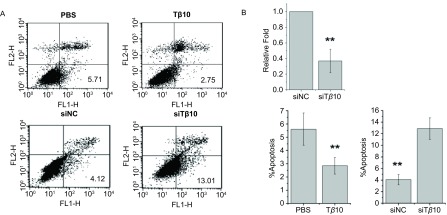
流式细胞分选检测T*β*10对A549细胞凋亡的影响。A：加入T*β*10能抑制A549细胞凋亡，干扰T*β*10则能促进A549细胞凋亡；B：Real-time PCR检测T*β*10的干扰效率。^**^P＜0.01。 Apoptosis rate of cell was detected after T*β*10 or siT*β*10 treatment by FCM assay. A: Add T*β*10 in A549 can inhibit the apoptosis rate, whereas transfection of T*β*10 siRNA can prompt apoptosis; B: Transfection efficiency of T*β*10 siRNA was detected by Real-time PCR. ^**^P < 0.01. FCM: flow cytometry.

### 2.2 Tβ10对A549细胞增殖和周期的影响

在A549细胞中加入100 ng/mL Tβ10，24 h后发现该组细胞增殖能力高于加入PBS的对照组。同时，转染siTβ10组与转染siNC组比，细胞的增殖能力则减弱，见图 2A。为了探究Tβ10对细胞增殖的调控是否和细胞周期的改变有关，我们使用流式单染观察各组细胞周期的变化。分析发现，加入Tβ10组G_2_期/M期，和S期的细胞比率增加，G_0_期/G_1_期的细胞数减少；与转染siNC组相比，转染siTβ10后G_2_期/M期和S期的细胞比率显著减少，G_0_期/G_1_期的细胞数增加（图 2B、图 2C）。以上结果说明Tβ10可以影响A549细胞周期，促进细胞增殖。

**2:**
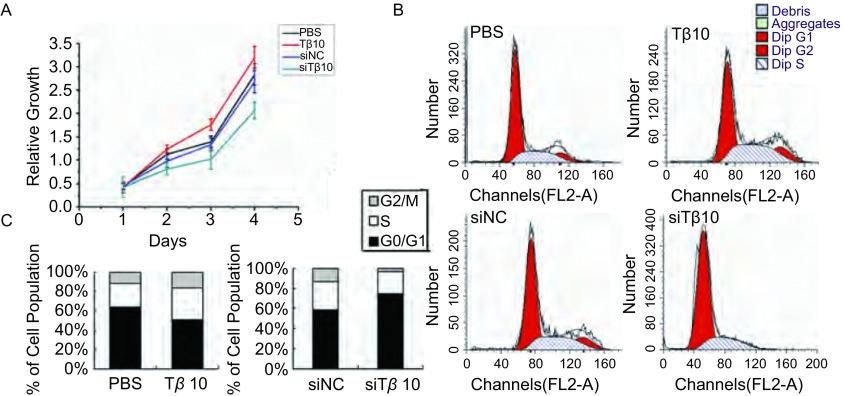
CCK-8分析及细胞周期分析检测T*β*10对A549细胞增殖及周期的影响。A：加入T*β*10能促进A549细胞增殖，干扰T*β*10则能抑制A549细胞增殖；B、C：加入T*β*10及T*β*10 siRNA后细胞周期发生了显著的变化。 The proliferation and cell cycle of cell was detected after T*β*10 or siT*β*10 treatment by cell was detected after T*β*10 and siT*β*10 treatment by CCK-8 assay and FCM assay. A: Add T*β*10 in A549 can prompt cell proliferation, whereas transfection of T*β*10 siRNA can inhibit cell proliferation; B, C: The cell cycle changed significently after T*β*10 or siT*β*10 treatment.

### 2.3 Tβ10对增殖和凋亡相关基因的影响

使用实时定量PCR筛查了一系列影响增殖和凋亡的基因发现，加入Tβ10能显著促进Cyclin A、Cyclin E的表达，抑制P53、Caspase-3的表达。反之，干扰Tβ10则能抑制Cyclin A、Cyclin E表达，促进P53、Caspase-3的升高，其他基因的变化均无统计学意义（图 3A）。随后我们用蛋白免疫印迹的方法验证了变化明显的几个基因的蛋白水平，加入Tβ10能显著促进Cyclin A、Cyclin E蛋白的表达，抑制P53、Caspase-3的表达；干扰Tβ10抑制Cyclin A、Cyclin E表达，促进P53、Caspase-3表达（图 3B）。这些结果提示Tβ10抑制细胞凋亡，促进细胞增殖可能是通过抑制P53、Caspase-3，促进Cyclin A、Cyclin E表达的方式实现的。

**3:**
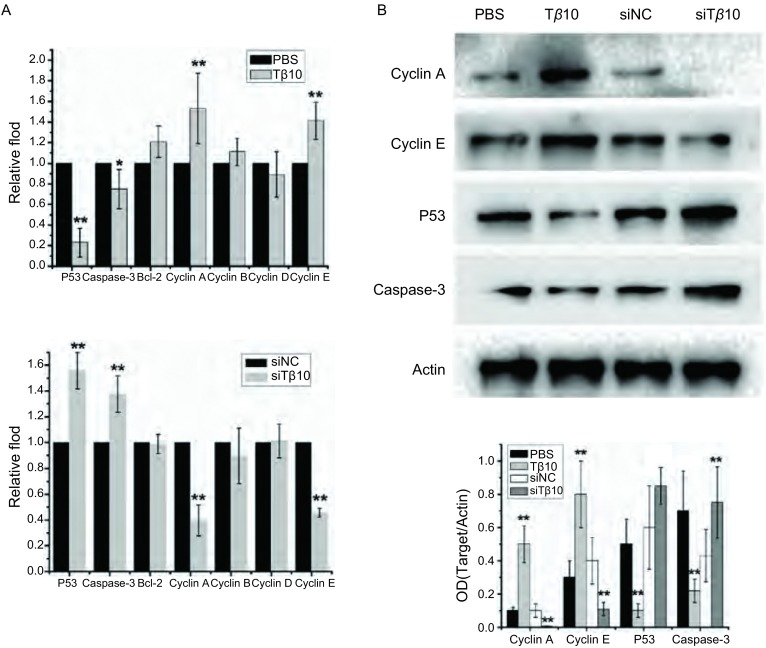
实时定量PCR及蛋白免疫印迹检测T*β*10对周期、凋亡相关基因的影响。A:加入T*β*10或干扰T*β*10后Cyclin A、Cyclin E、P53和Caspase-3 mRNA表达变化；B:加入T*β*10或干扰T*β*10后Cyclin A、Cyclin E、P53和Caspase-3蛋白变化情况。^**^P＜0.01。 The mRNA and protein level of Cyclin A, Cyclin E, Caspase-3 and P53 were detected by Real-time PCR and Western blot. A: The change of Cyclin A, Cyclin E, P53 and Caspase-3 mRNA level after add T*β*10 or transfection of T*β*10 siRNA in A549; B: The change of Cyclin A, Cyclin E, P53 and Caspase-3 protein level after add T*β*10 or transfection of T*β*10 siRNA in A549. ^**^P < 0.01.

## 3 讨论

目前，关于Tβ10在肿瘤中作用的研究主要集中在它对侵袭、转移的影响上。多篇文献^[5, 11-13]^报道，Tβ10能在甲状腺乳头状癌、肝癌、胆管癌等肿瘤中促进肿瘤的迁移、侵袭、转移。Tβ10与肿瘤细胞凋亡关系的报道较少，最新的研究^[14]^发现Tβ10可以通过上调ROS的水平促进卵巢癌细胞的凋亡。

在本研究中我们探讨了Tβ10对肺癌细胞凋亡的作用。根据文献^[6, 15]^报道，我们采用加入外源Tβ10的方法。发现加入Tβ10可以抑制肺腺癌细胞系A549细胞的凋亡并使在凋亡过程中起关键作用的酶Caspase-3的水平降低，而使用小干扰RNA干扰Tβ10则能促进A549细胞的凋亡并伴有Casepase-3的升高，这些结果提示Tβ10在肺癌中能起到抑制细胞凋亡的作用。肿瘤细胞能够抑制凋亡的机制有多种，P53蛋白表达的下调是其中重要原因。作为肿瘤抑制基因，*P53*的突变在肿瘤中是一个普遍的现象^[16]^，*P53*突变下调后能抑制DNA修复基因的激活导致肿瘤凋亡水平的降低^[17]^。在本研究中加入Tβ10抑制细胞凋亡的同时P53的作用可能是通过抑制P53的功能实现的。本研究Tβ10在肺癌细胞中能够抑制凋亡的结果和前人关于Tβ10在卵巢癌细胞凋亡中的作用正好相反，Tβ10在不同肿瘤细胞系中影响的凋亡相关通路也不尽相同。这些说明了在不同的细胞系中Tβ10所起的作用存在很大差异，需要进一步的研究来完善对Tβ10功能的准确认识。

本研究还发现Tβ10能影响肺癌细胞的增殖及细胞周期。加入Tβ10能促进细胞的增殖，同时增加G_2_期/M期和S期的细胞比率；反之，干扰Tβ10能降低细胞的增殖能力并增加处于G_0_期/G_1_期的细胞比率。细胞周期的变化和细胞周期素的含量有直接关系，通过检测发现，加入Tβ10能增加Cyclin A、Cyclin E mRNA及蛋白的表达水平，干扰Tβ10则能降低Cyclin A、Cyclin E mRNA及蛋白的表达水平。Cyclin A主要起到促进S期向G_2_期/M期转化，并在细胞增殖过程中起重要作用，而Cyclin E则为细胞从G_0_期/G_1_期进入S期的限速因子，且两者均为潜在的肿瘤标记物^[18]^。本研究的结果揭示了Tβ10对肺癌细胞的增殖促进作用，并提示Tβ10可能通过促进Cyclin A、Cyclin E的表达影响细胞周期，进而发挥它的促增殖作用。

综上所述，Tβ10具有调节细胞凋亡、增殖及细胞周期的功能。在肺癌细胞系中Tβ10能够通过抑制P53的表达抑制细胞凋亡，能够通过上调Cyclin A、Cyclin E的表达水平，促进细胞周期进程，促进细胞的增殖。抑制Tβ10能起到抑制细胞增殖、促进细胞凋亡的作用，Tβ10可能成为肺癌诊断的分子标记物及治疗靶标。
